# Enhanced surveillance of acute flaccid paralysis following importation of wild poliovirus in Xinjiang Uygur Autonomous Region, China

**DOI:** 10.1186/1471-2334-14-113

**Published:** 2014-02-27

**Authors:** Ning Wen, Chun-Xiang Fan, Jian-Ping Fu, Jing Ning, Yi-Xin Ji, Hui-Ming Luo, Hua-Qing Wang, Shuang-Li Zhu, Wen-Zhou Yu, Hai-Bo Wang, Hui Zhu, Fu-Qiang Cui, De-Xin Li, Shi-Wen Wang, Wen-Bo Xu, Li-Xin Hao, Ling-Sheng Cao, Li Luo, Lu Han, Lei Cao, Wei Xia, Xin-Qi Wang, Kathleen H Reilly, Fuerhati Wushouer, Sha-Sha Mi, Wei-Zhong Yang, Li Li

**Affiliations:** 1 Chinese Center for Disease Control and Prevention, 27 Nanwei Rd, Xicheng District, Beijing 100050, PR China; 2 The Center for Disease Control and Prevention of the Xinjiang Production and Construction Corps, 344 Wuxingnanlu Street, Urumqi city, Xinjiang Uygur autonomous region 830002, PR China; 3 Expanded Programme on Immunization, Xinjiang Uygur autonomous region Center for Disease Control and Prevention, 138 Jianquanyi Street, Urumqi city, Xinjiang Uygur autonomous region 830001, PR China; 4 WHO WPRO Regional Polio Reference Laboratory, National Institute for Viral Disease Control and Prevention, Chinese Center for Disease Control and Prevention, 155 Changbai Rd, Changping District, Beijing 102206, PR China; 5 Independent Consultant, New York City, NY, USA

**Keywords:** Acute flaccid paralysis, Wild poliovirus, Clinical compatible polio cases, China

## Abstract

**Background:**

After being polio free for more than 10 years, an outbreak occurred in China in 2011 in Xinjiang Uygur Autonomous Region (Xinjiang) following the importation of wild poliovirus (WPV) originating from neighboring Pakistan.

**Methods:**

To strengthen acute flaccid paralysis (AFP) surveillance in Xinjiang, “zero case daily reporting” and retrospective searching of AFP cases were initiated after the confirmation of the WPV outbreak. To pinpoint all the polio cases in time, AFP surveillance system was expanded to include persons of all ages in the entire population in Xinjiang.

**Results:**

Totally, 578 AFP cases were reported in 2011 in Xinjiang, including 21 WPV cases, 23 clinical compatible polio cases and 534 non-polio AFP cases. Of the 44 polio cases, 27 (61.4%) cases were reported among adults aged 15–53 years. Strengthening AFP surveillance resulted in an increase in the number of non-polio AFP cases in 2011 (148 children < 15 years) compared with 76 cases < 15 years in 2010. The AFP surveillance system in Xinjiang was sensitive enough to detect polio cases, with the AFP incidence of 3.28/100,000 among children < 15 years of age.

**Conclusions:**

Incorporating adult cases into the AFP surveillance system is of potential value to understand the overall characteristics of the epidemic and to guide emergency responses, especially in countries facing WPV outbreak following long-term polio free status. The AFP surveillance system in Xinjiang was satisfactory despite limitations in biological sample collection.

## Background

Poliomyelitis is a highly infectious disease caused by poliovirus. One in 200 infections can lead to irreversible paralysis. There is no cure for poliomyelitis, it can only be prevented through immunization of polio vaccine which is given multiple times and almost always protects a child for life. Poliomyelitis is one of the limited numbers of diseases that can be eradicated, as it only affects humans, an effective vaccine is available, and, moreover, immunity is lifelong. In 1988, the World Health Assembly resolved to eradicate poliomyelitis by 2000 [1,2]. Subsequently, the Global Polio Eradication Initiative reduced wild poliovirus (WPV) cases from an estimated 350,000 in 1988 to 650 reported cases in 2011, and indigenous transmission of type 2 WPV had been interrupted globally since 1999 [3].

Poliomyelitis had been historically endemic and widespread in China and stopping WPV transmission had been pursued through a combination of routine immunization and supplementary immunization activities (SIAs) based on high quality surveillance. Eventually, the last case of poliomyelitis due to transmission of indigenous WPV occurred in China in September 1994, and the Western Pacific Region has been certified as polio free since 2000 [4,5]. However, given the risk of WPV importation from areas where indigenous WPV transmission has never been interrupted, it is important to guarantee high quality surveillance, as many previously polio free countries have been affected by WPV importation [5-8].

WPV surveillance is conducted through reporting and laboratory testing of fecal specimens for all cases of acute flaccid paralysis (AFP) among children <15 years of age. An AFP case is defined as a child < 15 years of age showing acute or sudden onset of flaccid paralysis in one or more limbs, or other suspected poliomyelitis in a person of any age [9]. Two key performance indicators are monitored for AFP surveillance: 1) AFP surveillance system should be sensitive enough to detect at least 1 case of non-polio AFP per 100,000 children <15 years of age; 2) ≥ 80% of AFP cases have adequate collection of two fecal specimens within 14 days of the onset of paralysis (≥ 24 hours apart) [10]. A WPV case was defined as an AFP case for whom a stool specimen tested positive for WPV by virology, while a clinical compatible polio case was defined as an AFP case for whom stool specimens were missing or tested negative for WPV, but was determined to be polio-compatible by the provincial Polio Expert Committee of China after the standard 60-day follow-up examination. WPV importation has been a continuous threat to China which had experienced WPV importation historically [11-13], as it shares borders with 2 of the remaining 3 countries that had never interrupted WPV transmission as of 2012. On August 25, 2011, an outbreak following importation of WPV originated from neighboring Pakistan was confirmed in Xinjiang Uygur Autonomous Region (Xinjiang), China, after being polio free for more than 10 years. To detect potential polio cases, AFP surveillance was intensified in Xinjiang after the confirmation of the WPV outbreak. In this report, we describe the performance of intensified AFP surveillance compared with previous surveillance methods, as well as the epidemiological characteristics of AFP cases in Xinjiang.

## Methods

### Organization of AFP surveillance in China

In 1991, an active system of AFP surveillance with a target population <15 years of age was initially established, limited to representative regions of China located in different parts of the country. In 1993, the surveillance was extended to the national level and was conducted by the Chinese Center for Disease Control and Prevention (China CDC) under the leadership of the Ministry of Health.

Every case of AFP <15 years of age or clinical diagnosed polio cases at any age are reported to the county CDC through telephone or written report card by hospital physicians. After receiving case reports, the staffs of the county CDC conduct an epidemiological investigation and collect two stool specimens from each case for virological investigation within 14 days since the onset of paralysis. Stool specimens are placed in refrigerated containers and subsequently forwarded to the provincial polio laboratory where viral isolation is performed on L20B and RD cell cultures, and viral isolates are identified by micro-neutralization assays. Poliovirus isolates are forwarded to the National Polio Laboratory where intratypic differentiation is performed by polymerase chain reaction–restriction fragment–length polymorphism (PCR) and by enzyme-linked immunosorbent assay (ELISA). All tests are processed according to the standard guidelines recommended by World Health Organization (WHO). The National Polio Laboratory is part of the WHO Global Polio Laboratory Network and is fully accredited by the WHO as a regional reference polio laboratory.

Comprehensive hospitals at the county-level and above, the neurology specialized hospital, children’s hospital, infectious disease hospital, and the comprehensive hospital of Traditional Chinese Medicine are included as active surveillance hospitals, in which active searching of AFP cases are conducted to detect under-reported cases every 10 days by the county CDC.

### Intensifying active surveillance of AFP cases in Xinjiang

On August 28, 2011, immediately after confirmation of the WPV outbreak, “zero case daily reporting” (daily reporting even if no AFP cases have been found) was initiated in hospitals at the township level and above targeting AFP cases of all age groups in southern Xinjiang (where WPV transmission was limited, including Hotan, Kashgar, Bayingolin, Kezilesukeer and Akesu) and Urumqi (the capital city in Xinjiang), and in hospitals at the county-level and above targeting AFP cases <15 years in other prefectures (Table 1) as suspected polio cases (determined by clinicians without laboratory testing results) were also identified among adults. In keeping with definitions used in previous polio outbreaks, age ≥ 15 years was used to define adults [14-16].

**Table 1:** The definition of AFP case for zero case daily reporting and retrospective searching

**AFP surveillance**	**Areas**	**Hospitals**	**AFP case definition**
Zero case daily reporting	Southern Xinjiang and Urumqi	Hospitals at township level and above	AFP cases at any age
	Other prefectures	Hospitals at county level and above	AFP case <15 years or suspected polio cases at any age
Retrospective searching	Southern Xinjiang and Urumqi	Hospitals at township level and above	AFP case <15 years old, suspected polio case at any age in 2010; AFP case at any age, paralyzed from January 1 to August 28, 2011
	Other prefectures	Hospitals at county level and above	AFP case <15 years old, or suspected polio case at any age, paralyzed from January 1, 2010 to August 28, 2011

Note: *AFP* acute flaccid paralysis.

### Retrospective searching of AFP cases in Xinjiang

Retrospective searches of AFP cases were launched on August 28, 2011 in Xinjiang, aiming to detect under-reported AFP cases who sought health care from January 1, 2010 to August 28, 2011. In every selected hospital, both outpatient and inpatient records were checked in the pediatric department, neurology department, internal medicine department and the infectious disease department. Retrospective searches were conducted in hospitals at the township-level and above for southern Xinjiang and Urumqi, and in hospitals at the county-level and above in the remaining prefectures in Xinjiang. Case definitions for different areas are shown in Table 1.

### Ethical consideration

Based on WHO guidelines of AFP surveillance for the objective of poliomyelitis eradication, AFP surveillance is routinely conducted by the China CDC and the target population was expanded to improve the ability of AFP surveillance to detect potential polio cases in Xinjiang. The study was approved by the Chinese Center for Disease Control and Prevention institutional review board.

### Statistical analysis

Statistical tests were performed using SAS 9.1 software (SAS Institute Inc, Cary, NC, USA). Comparisons between groups were conducted using chi-square and Fisher’s exact tests for categorical variables. A *p*-value of <0.05 was considered statistically significant for all analyses.

## Results

### AFP surveillance in Xinjiang

Totally, 578 AFP cases were reported in 2011 in Xinjiang, including 21 WPV cases, 23 clinical compatible polio cases and 534 non-polio AFP cases (Table 2). Of the 578 AFP cases, 28 were < 1 year of age, 80 between 1 and 4 years, 57 between 5 and 14 years, 224 between 15 and 39 years, and 189 were ≥ 40 years of age. Of the 534 non-polio AFP cases, 145 (27.2%) were diagnosed as Guillain Barré syndrome and 111 (20.8%) with hypokalemic periodic paralysis.

**Table 2:** OPV immunization history of AFP cases by classification of AFP cases and age in 2011 in Xinjiang

**Classification of AFP cases**	**Age (years)**	**No.**	**0 dose**	**1-2 doses**	**≥ 3 doses**	**Unknown**
			**N (%)**	**N (%)**	**N (%)**	**N (%)**
WPV cases	< 1	6	3 (50.0)	1 (16.7)	2 (33.3)	0 (0)
	1-4	4	0 (0)	1 (25.0)	3 (75.0)	0 (0)
	5-14	0	0 (0)	0 (0)	0 (0)	0 (0)
	15-39	10	2 (20.0)	1 (10.0)	0 (0)	7 (70.0)
	≥ 40	1	0 (0)	0 (0)	0 (0)	1 (100.0)
	Total	21	5 (23.8)	3 (14.3)	5 (23.8)	8 (38.1)
Clinical compatible polio cases	< 1	3	2 (66.7)	0 (0)	1 (33.3)	0 (0)
	1-4	3	0 (0)	1 (33.3)	2 (66.7)	0 (0)
	5-14	1	0 (0)	0 (0)	0 (0)	1 (100.0)
	15-39	14	1 (7.1)	1 (7.1)	1 (7.1)	11 (78.6)
	≥ 40	2	0 (0)	0 (0)	0 (0)	2 (100.0)
	Total	23	3 (13.0)	2 (8.7)	4 (17.4)	14 (60.9)
Non-polio AFP cases	< 1	19	2 (10.5)	7 (36.8)	8 (42.1)	2 (10.5)
	1-4	73	0 (0)	1 (1.4)	63 (86.3)	9 (12.3)
	5-14	56	1 (1.8)	1 (1.8)	38 (67.9)	16 (28.6)
	15-39	200	7 (3.5)	23 (11.5)	8 (4.0)	161 (81.0)
	≥ 40	186	16 (8.6)	2 (1.1)	1 (0.5)	167 (89.8)
	Total	534	26 (4.9)	34 (6.4)	118 (22.1)	356 (66.7)

Note: *OPV* oral attenuated poliovirus vaccine, *AFP* acute flaccid paralysis, *WPV* wild poliovirus.

**Figure 1:**
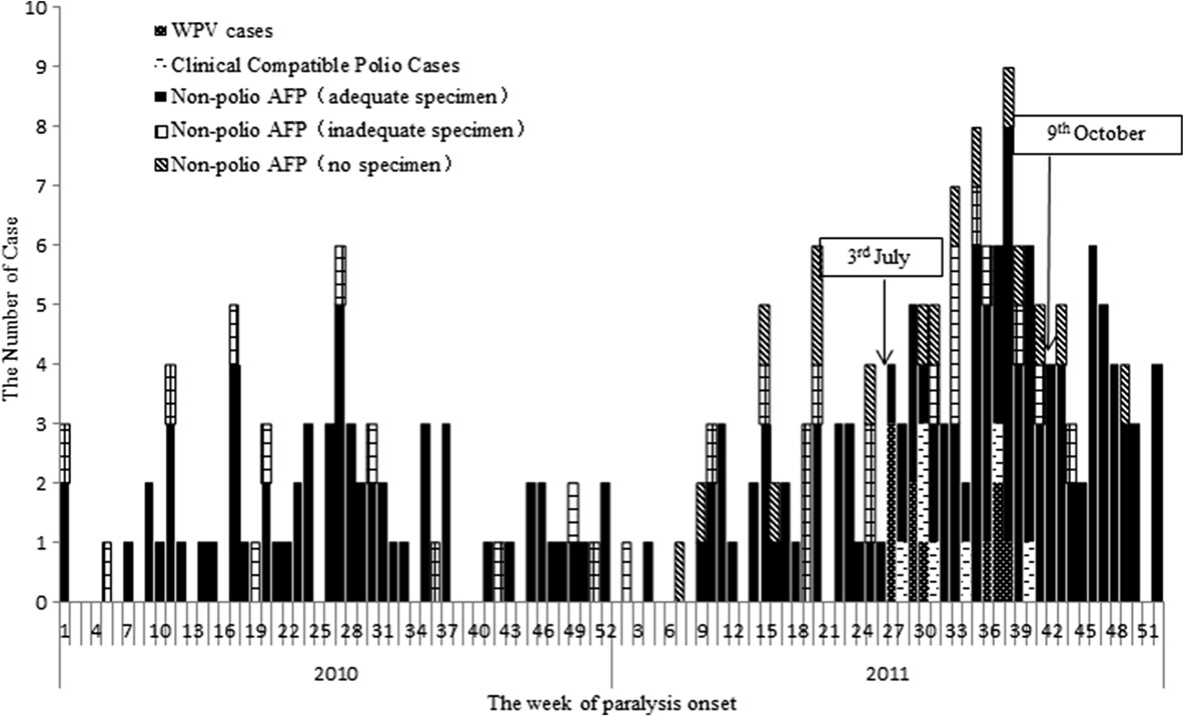
Weekly distribution of AFP cases < 15 years by classification and sample collection in 2010 and 2011 in Xinjiang.

Active surveillance and retrospective searches implemented after confirmation of the WPV outbreak, resulted in an increase in the number of non-polio AFP cases in 2011 (148 children < 15 years in 2011 compared with 76 cases <15 years in 2010) (Figure 1). Of the 148 non-polio AFP cases <15 years reported in 2011, 114 (77.0%) cases had adequate fecal specimens, 18 (12.2%) cases had inadequate specimens, and 16 (10.8%) cases did not provide fecal specimens; while in 2010, 63 (82.9%) cases had adequate fecal specimens and 12 (17.1%) cases had inadequate specimens.

**Figure 2:**
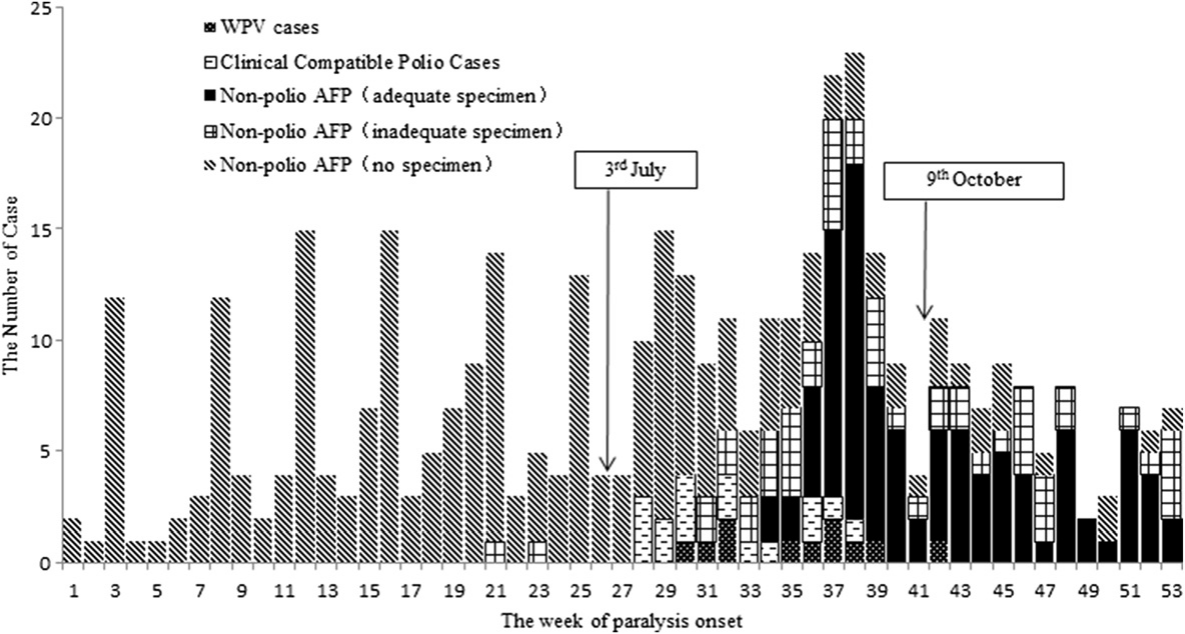
Weekly distribution of AFP cases ≥ 15 years by classification and sample collection in 2011 in Xinjiang.

Of the 386 non-polio AFP cases ≥ 15 years reported in 2011, 98 (25.4%) cases had adequate fecal specimens, 51 (13.2%) cases had inadequate specimens and 237 (61.4%) cases did not provide fecal specimens (Figure 2). Specimens were not collected among almost all AFP cases ≥ 15 years before the confirmation of WPV outbreak. However, of the 158 non-polio AFP cases who were paralyzed after the confirmation of the WPV outbreak, 94 (59.5%) cases had adequate fecal specimens, 36 (22.8%) cases had inadequate specimens, and 28 (17.7%) cases did not provide fecal specimens.

### WPV and clinical compatible polio cases

**Figure 3:**
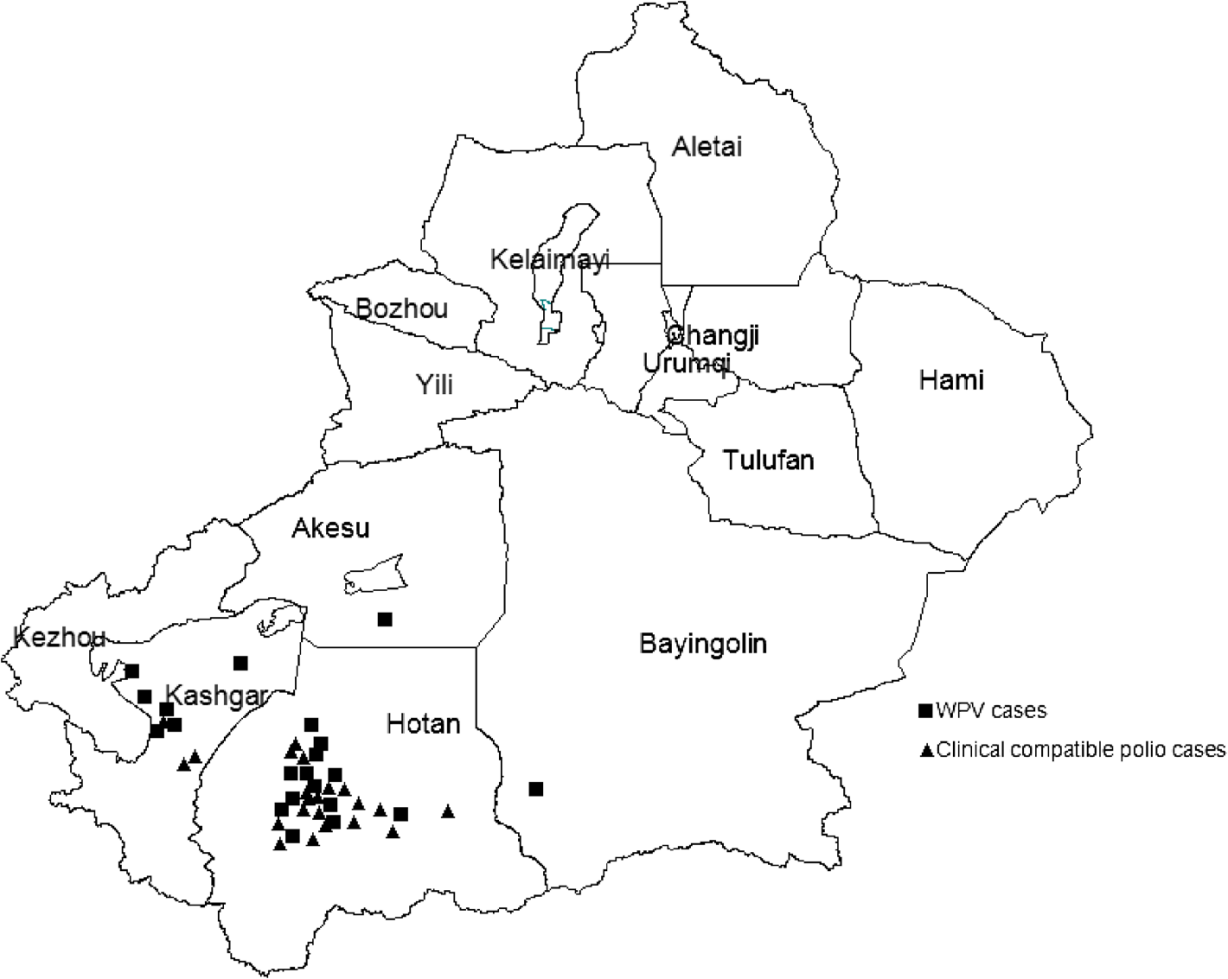
**Distribution of laboratory confirmed WPV cases and clinical compatible.** Polio Cases in Xinjiang in 2011.

WPV and clinical compatible polio cases were limited to four southern prefectures (Figure 3): Hotan (13 WPV cases and 19 clinical compatible polio cases), Kashgar (6 WPV cases and 3 clinical compatible polio cases), Bayingolin (1 WPV case), and Akesu (1 WPV case and 1 clinical compatible polio case). The first WPV case was paralyzed on July 3, while the last WPV case was paralyzed on October 9. The first clinical compatible polio case was paralyzed on July 5, while the last clinical compatible case was paralyzed on October 4. The first adult WPV case was paralyzed on July 22, and the first adult clinical compatible polio case was paralyzed on July 5. Six clinical compatible polio cases were paralyzed after the confirmation of the WPV outbreak, in which 2 cases were younger than 15 years of age.

Based on the population in the four southern prefectures, WPV and clinical compatible polio cases incidence were 0.22/100,000 and 0.24/100,000, respectively, both with highest incidence among children <1 year (3.46/100,000 and 1.15/100,000, respectively), followed by children aged 1–4 years (both 0.64/100,000). No WPV cases were reported among children aged 5–14 years, and only 1 clinical compatible polio case was reported. More than half (27/44 (61.4%)) of polio cases were reported among adults aged 15–53 years. The WPV incidence among males (0.29/100,000) was higher than among females (0.15/100,000), as was the incidence of clinical compatible polio cases among males (0.41/100,000) compared with females (0.06/100,000).

### Oral attenuated poliovirus vaccine (OPV) immunization history of AFP cases

Of the 578 AFP cases reported in 2011, only 127 (22.0%) cases received ≥ 3 doses OPV, and 378 (65.4%) cases did not have detailed OPV immunization histories, of which 349 (92.3%) were adults cases. Totally, 70.9% (117/165) of cases among children received ≥ 3 doses OPV and 4.9% (8/165) did not receive any OPV doses. Among AFP cases < 1 year of age, 3 (50.0%) WPV cases, 2 (66.7%) clinical compatible polio cases and 2 (10.5%) non-polio AFP cases did not receive any OPV doses. Among the 200 AFP cases with known immunization histories, there were significantly more non-polio AFP cases that received ≥ 3 doses OPV than polio cases (including WPV cases and clinical compatible polio cases: 66.3% (118/178) and 40.9% (9/22), respectively; *P* = 0.02).

### Performance of AFP surveillance system in Xinjiang

The reported non-polio AFP incidence in Xinjiang was >1 per 100,000 children <15 years of age during 2006–2010, but the collection rates of adequate stool specimens were lower than 80% in 2008 and 2009. After the comprehensive strengthening of the AFP surveillance system, the reported incidence of AFP (3.28/100,000) among children <15 years of age in Xinjiang in 2011, was nearly twice more than that in 2010 (1.63/100,000). Most importantly, there were also increases for system sensitivity in southern Xinjiang: Hotan 0.82/100,000 *vs.* 3.40/100,000, Kashgar 1.86/100,000 *vs.* 4.34/100,000, Akesu 1.20/100,000 *vs.* 2.76/100,000, and Bayinguole 1.25/100,000 *vs.* 4.48/100,000. In 2011, the reported incidences of AFP were higher than 2/100,000 (target required by WHO for polio epidemic countries) among children < 15 years of age in all but Yili Prefecture.

However, in 2011, some timely indicators of AFP surveillance system at the provincial and prefectural levels did not meet the target required by the Ministry of Health and WHO. The proportion of AFP cases with 2 adequate stool specimens collected within 14 days of paralysis onset did not meet the target of ≥ 80% in Xinjiang (77.0%), Hotan (52.9%) and Kashgar (76.7%).

## Discussion

This report documents the performance of an intensified AFP surveillance system in Xinjiang following WPV importation from neighboring Pakistan. Totally, 578 AFP cases were reported in 2011 in Xinjiang, including 21 WPV cases and 23 clinical compatible polio cases. Based on active surveillance and high quality retrospective searches, the number of non-polio AFP cases reported in Xinjiang in 2011 was nearly twice more than that in 2010. This was a limited transmission of WPV in Xinjiang, as WPV cases and clinical compatible polio cases were confined to four southern prefectures: Hotan, Kashgar, Bayingolin, and Akesu. Children less than 5 years of age were most likely exposed and vulnerable to the virus, however, adults who accounted for more than 50% of polio cases may be an important source of infection for children. For timely detection of adult polio cases, adults with AFP were also incorporated into the AFP surveillance system in Xinjiang.

The primary objective of AFP surveillance is to detect, investigate, report and promptly implement an emergency response. Detecting and investigating all cases of non-polio AFP in the children <15 years of age are among the most important criteria for polio free certification. In recent years, the reported incidence of non-polio AFP cases was efficient and even exceeded the WHO-established minimum target of 1 non-polio AFP case per 100,000 children. However, the number of non-polio AFP cases in Xinjiang doubled after active AFP surveillance was strengthened following WPV importation in 2011 compared with 2010. Therefore, although routine AFP surveillance in polio free countries usually meets the WHO-established requirement, in order to obtain prompt reports of AFP cases after WPV is imported and results in onward transmission, intensified active AFP surveillance and retrospective searches should be conducted. Another important criterion of AFP surveillance is adequate collection of two stool specimens within 14 days of paralysis onset. The indicator in 2011 did not meet the target of ≥ 80% in Xinjiang (77.0%), Hotan (52.9%) and Kashgar (76.7%). Moreover, 6 clinical compatible polio cases were reported after active AFP surveillance was strengthened following WPV importation, in which there were 2 cases <15 years of age. These cases were classified as compatible because of inadequate stool collection, therefore, the potential factors contributing to inadequate stool collection should be identified and addressed so that AFP surveillance system can be improved by minimizing the time between paralysis onset, case notification, investigation and sample collection. In order to achieve the optimal standards recommended by WHO, both in case reporting and in the collection of stool specimens, greater attention and involvement of clinicians and other health workers is needed [17].

Adults aged 15–39 years accounted for more than 50% of polio cases, although with relatively low incidence. Any country with no recent WPV transmission may face similar outbreaks characterized by a large proportion of cases in older age groups. [7,16,18-21] It was found that antibody seroprevalence decreased with increasing age [22], and seroprotection rates and GMTs were low in both adolescents and adults [23]. In order to detect all the polio cases in time and interrupt the potential transmission of WPV, adult AFP cases were also incorporated into AFP surveillance system in Xinjiang after the confirmation of the WPV outbreak. As far as we know, this was the first time that adult AFP cases were monitored with the same WHO requirement as child AFP cases. This was found to be a useful way to understand the overall epidemic and to discover the index case. After index polio cases were detected, a series of epidemiological investigations and emergency response activities were implemented to prevent WPV transmission, such as serological surveys, poliovirus surveillance for close contacts of AFP cases and healthy children, environmental surveillance, cleaning and disinfection of toilets used by polio cases, and SIAs [24]. In addition to polio cases, WPV was also isolated from healthy children, close contacts of AFP cases, and environmental samples collected in Kashgar and Hotan prefectures. WPV strains were isolated in Beijing among 3 healthy students from Hotan prefecture [24]. Based on the findings from this 2011 AFP surveillance in Xinjiang, the target population for SIAs was expanded to <40 years in southern Xinjiang. Five rounds of SIAs were conducted in Xinjiang from August 2011 to April 2012, with a total of 43.7 million doses of OPV administered, which led to the quick restriction of WPV transmission [24]. The incidence of polio cases was higher among males than females (WPV incidence: 0.29/100,000 *vs.* 0.15/100,000; incidence of clinical compatible polio cases: 0.41/100,000 *vs.* 0.06/100,000). It has been observed that clinical manifestations of poliovirus are more common among males than females [25], even though males and females share similar vaccination coverage. There are two possible assumptions as to why males had higher polio incidence than females: among Uygur minorities, males may have more opportunities to participate in public activities, which puts them at higher risk of infection and women in the young adult age groups may have additionally benefitted from exposure to excreted OPV while caring for the young [26].

It was not surprising that more non-polio AFP cases received ≥ 3 doses OPV than polio cases. OPV induced immunity had been well proven, OPV has been the main tool in the WHO polio eradication program and many countries have achieved polio eradication by OPV immunization. In 2011, only 70.9% (117/165) children AFP cases received ≥ 3 doses OPV, and 4.9% (8/165) did not receive any OPV doses in Xinjiang. The reported number of OPV doses of non-polio AFP cases can be used as a proxy measure of OPV coverage [27]. A serological study was conducted to estimate poliovirus antibody seroprevalence in 2010, immediately prior to the 2011 WPV importation into Xinjiang [22]. Almost all (95.8%) children <15 years of age in Xinjiang were seropositive to type 1 poliovirus, which is comparative to the estimation of OPV coverage by AFP surveillance system that 4.9% of non-polio AFP cases <15 years of age were not immunized in 2011. The low rate of full OPV immunization (≥3 doses) among child AFP cases which suggests a gap in immunization status or the presence of parts of population not immunized, together with immigrants and emigrants from and to Pakistan, may lead to another WPV outbreak in Xinjiang.

## Conclusions

Incorporating adult AFP cases into AFP surveillance system is of potential value to understand the overall characteristics of the epidemic and to guide emergency response, especially in countries facing WPV outbreak following long-term polio free status. Case reporting by AFP surveillance and subsequent laboratory diagnosis were essential to evaluate the interruption of WPV circulation. The AFP surveillance system in Xinjiang was satisfactory despite limitations in biological sample collection. In order to achieve the optimal standards recommended by WHO, both in case reporting and in the collection of stool specimens, greater attention and involvement of clinicians and other health workers is needed.

## Competing interests

The authors declare that they have no competing interests.

## Authors’ contributions

Authors NW, C-XF, HL, H-QW, W-Z Y, H-BW, F-QC, W-ZY and LL designed investigation proctol of outbreak. NW, C-XF, J-PF, JN, HL, H-QW, W-ZY, H-BW, F-QC, L-XH, L-SC, LC, WX, X-Q W, LL, LH, FW and S-SM conducted epidemiological investigations and emergency response, including AFP surveillance, specimen collection, SIAs etc. NW and C-XF were responsible for national AFP surveillance and data quality control. Y-XJ, S-LZ, HZ, DXL, S-WW and W-BX were responsible for laboratory testing of serum and stool specimens. H-B Wang performed all statistical analysis. H-BW, KHR, NW and C-XF wrote the first draft of the manuscript. All authors contributed to and have approved the final manuscript.

## Authors’ information

Ning Wen, Chun-Xiang Fan, Jian-Ping Fu, Jing Ning and Yi-Xin Ji are co-first authors.

## Pre-publication history

The pre-publication history for this paper can be accessed here:

http://www.biomedcentral.com/1471-2334/14/113/prepub
